# Investigating Plastic–Metal Interactions in Aquatic Environments Using Laser Ablation ICP–MS and Chemical Markers

**DOI:** 10.1021/acsestwater.5c01387

**Published:** 2026-02-18

**Authors:** Davide Spanu, Ludovica Botta, Stefano Carnati, Tommaso Grande, Gabriela Kalčíková, Luca Nizzetto, Andrea Pozzi, Luka Šupraha, Gilberto Binda

**Affiliations:** † Department of Science and High Technology, University of Insubria, Via Valleggio 11, Como 22100, Italy; ‡ Department of Theoretical and Applied Science, University of Insubria, Via J.H. Dunant 3, Varese 21100, Italy; § Faculty of Chemistry and Chemical Technology, 112794 University of Ljubljana, Večna Pot 113, Ljubljana 1000, Slovenia; ∥ Faculty of Mechanical Engineering, Brno University of Technology, Technická 2896/2, Brno 61669, Czech Republic; ⊥ Norwegian Institute for Water Research (NIVA), Økernveien 94, Oslo 0579, Norway; # Research Centre for Toxic Compounds in the Environment, Masaryk University, Kamenice 753/5, Brno 625 00, Czech Republic

**Keywords:** pollution, aging, biofouling, sorption, additives, environmental risk, plastisphere

## Abstract

Plastics in aquatic environments interact with metals, influencing their fate and transport. Biotic aging of plastics plays a pivotal role in this process, but the mechanisms are still unclear. Here, we employed laser ablation–inductively coupled plasma mass spectrometry (LA–ICP–MS) to track elemental cross-sectional distribution in biotically aged plastics and assess metal enrichment within the biofilm. Copper, sorbed from the water environment, was used as a marker of biofilm presence, while antimony and tin marked the plastic phase for polyethylene terephthalate (PET) and polylactic acid (PLA), respectively. Aged samples revealed distinct metal distribution patterns tracking copper enrichment on the surface, whereas physicochemical changes happened on the plastic surface after aging, highlighting the biofilm presence. Copper depletion in water during the aging experiment confirmed that aged plastics accumulate this metal, showing the key role of biofilms in governing this process. Conventional analysis based on acid digestion of plastic fragments only partially captured this enrichment, underscoring the added value of LA–ICP–MS to specifically track metals accumulated from the water in comparison to those present in the polymer matrix. These results highlight the need to account for biofilm-mediated processes in risk assessments and establish LA–ICP–MS as a powerful tool for investigating metal–plastic interactions.

## Graphical abstract

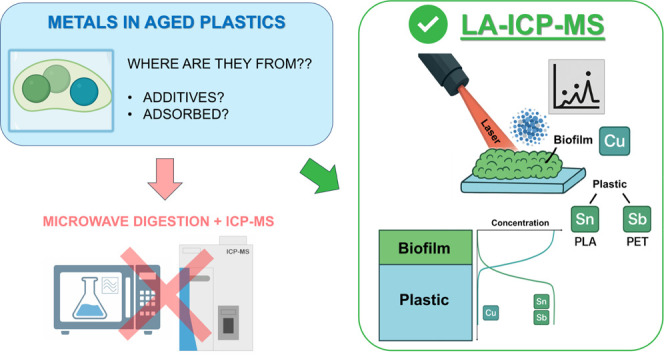

## 1 Introduction

The interaction between plastic and (trace) elements in waters has attracted the interest of researchers: the possible implications, such as the alteration of natural biogeochemical cycling and the potential vector effect of toxic metals through the trophic chain, are worrying for ecosystem health. Their environmental relevance, however, is yet to be understood.^1–5^

Plastic has, in fact, been observed to actively accumulate metal ions from the environment in water bodies. The processes of plastic aging (i.e., the degradation and alteration of plastics by abiotic and biotic factors) play a pivotal role here.^2,6,7^ In particular, biotic aging has been identified as a key determinant of the metal adsorption processes:^3^ the colonization of plastic by a microbial community can completely alter the surface properties of plastic, providing different surface functional groups and altering surface hydrophobicity, increasing metal sorption from the environment.^8^ In addition, several microorganisms are known to actively accumulate metal ions inside their cells as some of them are essential for their life.^9^ As an example, Cu is an essential element (at environmentally relevant concentrations), and microorganisms can both internalize it in their cell tissues or immobilize it in the extracellular exudates, such as polysaccharides.^10–12^

In addition to adsorbing metals from the environment, plastics can contain several inorganic chemicals, such as functional additives, pigments, and impurities.^4,13,14^ Other metals are instead added as a catalyst during the production of specific polymers: a known example is the use of Sb in polyethylene terephthalate (PET) to favor the polycondensation of this polymer.^4,15^ These additives may leach out from the plastic matrix into the surrounding water, and the environmental aging of plastics appears to further influence these leaching processes. This further complicates the evaluation of potential risks related to plastic pollution.^16,17^

A crucial aspect of studying the interaction between plastics and metals is distinguishing between additives incorporated within the polymer matrix and metals accumulated from the environment. To address this knowledge gap, several studies have investigated environmental samples through bulk analysis (e.g., through acid digestion), as well as integrating specific leaching solutions.^18–20^ Within this framework, laser-based techniques, such as laser ablation—inductively coupled plasma—mass spectrometry (LA–ICP–MS), are emerging as tools to resolve the microscale spatial distribution of both inorganic additives and trace elements sorbed from the environment.^21^ Some recent applications showed the potential of LA–ICP–MS to track elemental content in pristine and aged plastic samples, including environmental samples.^22–25^ While the interest in these techniques is increasing, there still are issues hampering a broader applicability of LA–ICP–MS to understand the interaction dynamics of metals and plastics, such as the complex and poorly known array of metal-containing additives present in plastics and the several processes affecting metal adsorption in natural environments (e.g., climate, pH, and particulate matter).^18,19,21,26^

In this study, we employed a LA–ICP–MS-based approach to trace the enrichment of trace metals on plastic materials aged in synthetic freshwater to allow biofilm growth. Laboratory-scale experiments simulating plastic biotic aging help in reducing ambiguity for this kind of experiment, while chemical markers can track the polymer matrix, allowing a detailed cross-sectional view of metal adsorption from the environment. Here, we used plastic-specific inorganic additives as valid markers to detect the polymer matrix (e.g., Sb in PET and Sn in PLA, present as catalyst residuals after polymer production^13,16,24,27^ ). These elements are in fact present in the plastic products at easily detectable concentrations (in the tens of mg/kg range). To observe the unique help of chemical markers in investigating plastic–metal interactions, we also investigated a marker-free plastic sample made of polypropylene (PP) in this experiment. We also monitored Cu concentration in plastic and in water as this trace element is known to enrich in biofilms.^28–30^ By combining physicochemical characterization, total metal content analysis, and scanning electron microscopy, we investigated the biofilm thickness on plastic surfaces and their capacity to selectively accumulate elements from the surrounding water. Implementing these approaches, we aim to improve understanding of plastic–metal interaction dynamics and to demonstrate the potential and limitations of LA–ICP–MS for monitoring and profiling of metals at the plastic–biofilm interface. This investigation also sheds light on the key implications of biotic aging in affecting the properties of plastic pollutants.

## 2 Materials and Methods

### 2.1 Reagents and Solutions

All solutions were prepared using ultrapure water (18.2 MΩ x cm resistivity) obtained from a Sartorius (Germany) Arium pro VF. If not differently specified, labware and plastic samples were rinsed with ultrapure water. Synthetic freshwater (i.e., algal growth medium) was prepared following the Z8 formulation.^31^ Nitric acid for labware washing and sample acidifications was obtained by dilution of HNO_3_ Suprapur (Sigma-Aldrich, United States of America). Commercial H_2_SO_4_ (Analytika, 95% v/v pure) and H_2_O_2_ (Fisher Chemical, 30% v/v for trace analysis) were instead used as purchased. FeSO_4_·7 H_2_O, D-(+)-glucose (≥99.5% in purity), and phenol (≥99% in purity) were instead purchased from Sigma-Aldrich, United States of America.

All LDPE containers used for laboratory purposes were subjected to a two-step cleaning protocol involving ultrapure water, NALGENE solution, and a 2% v/v HNO_3_ solution. Erlenmeyer flasks in glass that were instead used for the aging were autoclaved and rinsed with ultrapure water.

Prior to each digestion cycle for the analysis of metals in plastic fragments, PTFE vessels were also pretreated through a two-step process: they underwent an initial heating cycle with 65% HNO_3_, followed by three rinses with ultrapure water, and were then conditioned with the same acid mixture applied during digestion.^32^

### 2.2 Plastic Samples and Aging Experiment

Plastic samples were obtained from commercial objects in PP, PET, and PLA by cutting “confetti-like” fragments from single-use transparent food containers (single-use PP lids from Pro-Pac, Germany, single-use PET bottles from Flaschenland, Germany, and single-use PLA lids from PAPSTAR, Germany) in squares of about 5 × 5 × 0.3 mm for PP and PLA and about 5 × 5 × 0.6 mm for PET.

All the aging treatments were performed in 100 mL glass Erlenmeyer flasks, autoclaved and rinsed with ultrapure water prior to the experiment, and performed in three replicate batches. Then, 100 mL of growth media (prepared by diluting Z8 growth media at a final 10% v/v concentration in ultrapure water) was added to the flasks. After that, 500 μL of an algal community inoculum isolated from a pond in Oslo, Norway (59.9227 °N, 10.7955 °E, main physicochemical features listed in Table S1), was added to all flasks. Treatments were obtained by adding 15 plastic fragments of a given polymer type in individual flasks (reaching a total mass of in the different batches weight of 256 ± 47 mg for PET, 158 ± 59 mg for PLA, and 109 ± 26 mg for PP, expressed as average ± standard deviation). An algal growth positive control was included: this is represented by flasks with medium and inoculum but with no addition of plastic fragments. This was used to verify the content of dissolved Cu without the effect of plastics and their biofilms. The flasks were then left at 15 °C and 60 μmol m^2^/s light radiation in the 400–700 nm range (measured with a Skye SpectroSense2 + system, United Kingdom) for 3 weeks, a sufficient time for the development of biofilm communities on plastic.^28,33^ Flasks were shaken and moved every 24 h to ensure a random variability of light irradiation and temperature conditions. Water samples were collected weekly for the measurements of dissolved metal markers (i.e., Sb and Sn for potential release of additives from plastic and Cu to observe the potential adsorption of biofilms). Plastic samples were collected and analyzed only at the end of the experiment.

### 2.3 Physicochemical Characterization of Plastic and Biofilms

After collection, all plastic samples were air-dried for 24 h and analyzed for their physicochemical properties to assess the changes induced by biotic aging. In addition, specific measures to analyze the physicochemical features of the biofilm community were investigated too.

To observe the changes in surface functional groups, we used Fourier transformed infrared spectroscopy in the attenuated total reflection (ATR-IR) mode (Nicolet iS 10, Thermo Scientific, Waltham, MA, United States of America). Thirty two scans were performed for every sample in the 4000–650 cm^–1^ spectral interval, with a resolution of 0.482 cm^–1^. A background spectrum was recorded prior to every measurement. Collected IR spectra were then normalized on the maximum absorbance peak using Origin 2024 pro software (OriginLab Corporation).

Scanning electron microscopy (SEM) images were collected to examine the surface micromorphology of plastic fragments with and without the presence of a biofilm as well as to understand the quantity of the ablated plastic material during the LA–ICP–MS analyses (see Section 2.6) using a Philips (Netherlands) field emission gun-scanning electron microscope (FEG-SEM), with a 20 keV beam under high vacuum conditions. Before SEM analysis, samples were made more conductive by covering them with a 5 nm thick gold layer using a Cressington (UK) 108 auto vacuum sputter coater.

Static water contact angles were also measured to assess the hydrophobicity of plastic samples using a 3D printed instrument.^34^ Briefly, 3 μL of ultrapure water was deposited on the sample surface, and pictures were collected via a smartphone (Samsung Galaxy S21FE) camera. Then, contact angles were computed on the collected images using ImageJ software using the “drop_analysis” plugin.^35^ Samples were collected in triplicate for each specimen.

The total mass of the biofilm on the aged plastic fragments was estimated after oxidation with a Fenton reagent.^33,36^ The oxidation was performed with three replicates containing approximately 20 mg of plastic each. 0.5 mL of Fe^2+^ solution (15 g/L FeSO_4_·7 H_2_O with 6 mL/L 97% (v/v) H_2_SO_4_) and 0.5 mL of 30% (w/w) H_2_O_2_ were added, and oxidation was carried out overnight at room temperature (22 ± 2 °C). The samples were then removed from the solution, dried to constant mass at room temperature (22 ± 2 °C), and weighed again. The mass loss after oxidation was then estimated as the biofilm biomass, applying the following equation

$$B=\frac{m_{pre}-m_{post}}{m_{pre}}\;\%$$

where *B* indicates the amount of the biofilm on aged plastics (as a percentage of the total mass), *m*_pre_ is the mass of the fragments before oxidation (mg), and *m*_post_ is the mass of the aged MPs after oxidation (mg). The mass loss induced by Fenton oxidation was assessed for pristine plastic samples, too, in order to observe potential polymeric degradation.

The content of extracellular polymeric substances (EPS) of the biofilm developed of plastic was then assessed.^33,36^ This parameter can be used as an index of biomass and represents the fitness of microorganisms attached.^37^ 1.5 mL of ultrapure water was added to approximately 25 mg of dried aged MPs (three replicates). The samples were incubated for 30 min at 80 ± 1 °C and filtered after cooling to room temperature (0.45 μm pore size, VWR Avantor, United States of America). 1 mL of the filtrate was added to 0.5 mL of 6% (*w/v*) phenol and 2.5 mL of H_2_SO_4_ (97% (v/v)). After cooling to room temperature, the absorbance was measured at 490 nm using a Spectramax iD3 (Molecular Devices, United States of America). D-(+)-glucose was used to prepare the calibration curve, and the EPS content was expressed as milligrams of EPS per gram of plastic. Absorbance values of the solution after the extraction of pristine plastic were used as blanks.

### 2.4 Analysis of Total Metal Content in Plastic

The total metal content of plastic samples was assessed using a microwave-assisted acid digestion protocol set up by our research team (please also refer to this publication for detailed methods and quality protocols).^32^ Plastic sample digestion was obtained through microwave-assisted heating in a closed system (ETHOS One, Milestone MLS, United States of America) equipped with polytetrafluoroethylene (PTFE) vessels. Briefly, approximately 100 mg was weighed and inserted into each vessel, and 4 mL of 65% v/v HNO_3_ and 1 mL of 95% v/v H_2_SO_4_ were added to the vessels. The materials were then digested by applying a temperature ramp, reaching 200 °C for 45 min. A further digestion was then performed by adding 0.1 mL of H_2_O_2_ to each vessel and leaving to react for 30 min at room temperature, after which another H_2_O_2_ addition of 0.1 mL was made. Solutions were then left at room temperature inside the vessels until cool.

### 2.5 Instrumental Analysis of Metals in Solution

Solutions obtained by the different aging batches and after acid digestions were then analyzed for their content of Sn, Sb, and Cu using an ICP–MS instrument. All these solutions were filtered (0.45 μm PTFE filter), acidified with HNO_3_ reaching a 2% v/v concentration, and spiked with two internal standards (Rh and Re, respectively). The solution was then finally analyzed with a Thermo Scientific ICAP Q (United States of America) ICP–MS, and metal­(loid) quantification was obtained by external calibration. Details of the instrumental setup are listed in Table S2.

### 2.6 Depth Profiling of Metal Content in Plastic

The spatially resolved contents of Sb, Sn, and Cu in the plastic samples were determined using a LA–ICP–MS approach: our system is based on a custom-made laser ablation cell previously developed by our research group, which combines laser ablation instrumentation (UP 266, New Wave Research, Fremont, CA, United States of America) with a Nd: YAG laser source (266 nm pulse 3–4 ns) and a Thermo Fisher IcapQ (United States of America) ICP–MS.^38,39^

After a preliminary setup of conditions (see the detailed results in Section 3.3) for the working parameters to obtain a reliable ablation of the plastic polymers, we selected the working conditions for ICP–MS listed in Table S2.

To perform depth profiling of metals, we randomly selected 2 spots on the plastic fragment surface, and we then performed 5 consecutive ablations (single shots with 120 μm spot size and 6.7 J/cm^2^ fluency) as applied in other settings to environmental plastics.^22^ The transport of the ablated material to the ICP–MS instrumentation was then guaranteed by a He flow rate of 1 L/min.

The following *m*/*z* channels were monitored: ^13^C, ^63^Cu, ^118^Sn, and ^121^Sb. All signals were collected using the ICP–MS standard mode that avoids the use of the collision cell. All peak areas were estimated using Origin 2024 pro software (OriginLab Corporation) and then analyzed as the ratio with the ^13^C isotope (internal standard) to ensure reliable signals of the ablated plastic amount.^22,40,41^

Crater depth was estimated by SEM by focusing sequentially on the rim and on the bottom of the crater and taking the difference in the working distance as the depth value.

### 2.7 Data Treatment

For all concentration data from water samples and digested plastics, limits of detection (LODs) and limits of quantification (LOQs) were obtained after the analysis of 5 procedural blank samples and were calculated as 3 times the standard deviation of the blanks for LODs and as 10 times for LOQs.

All data sets were evaluated for normality using a Shapiro–Wilk test prior to further analysis. As the samples showed a normal distribution among replicates, *t* tests were performed to compare the trends in physicochemical features and metal content in pristine and aged plastics as well as to evaluate differences in Cu depletion of time in water. The threshold for statistical difference was established as *p* < 0.05.

## 3 Results and Discussion

### 3.1 Impact of Biotic Aging on Plastic Physicochemical Properties

Biotic aging affected the physicochemical properties of all of the plastic samples. However, the extent of these alterations was strictly polymer-dependent. As a primary effect, the coating of the biofilm evidently influenced the surface morphology of plastic (Figure S1). SEM micrographs (Figure 1) show a thick layer of microorganisms homogeneously covering the plastic samples after the aging, which varied from about 5 μm up to about 50 μm depending on the plastic type. Such a difference in thickness may be induced by both plastic polymer hydrophobicity and micromorphology and the type of chemicals present as additives. In this study, PP and PLA showed comparable thickness, while PET showed a distinct lower thickness. This evidence is corroborated by both a significantly lower mass of the biofilm observed after Fenton digestion and a lower concentration of EPS in the PET-aged fragments in comparison with the samples made of other polymers (Figure S2). This may be induced by a lower hydrophobicity, potentially reducing microbial attachment,^28^ the limited availability of carbon as a source for bacteria being a nondegradable polymer,^42^ and the relatively high content of additives, such as Sb, which may potentially hamper a quick attachment of microorganisms on the polymeric substrates.^43^ Observing the microbial community composition from SEM micrographs, several filamentous algae and coccoids were evident, as well as several residuals of diatom frustules (Figure S3b), as indicators of widespread colonization by microorganisms in these samples.^19,44^ The pristine samples, on the other hand, show a smooth surface with only minor defects (Figure S3).

**Figure 1:**
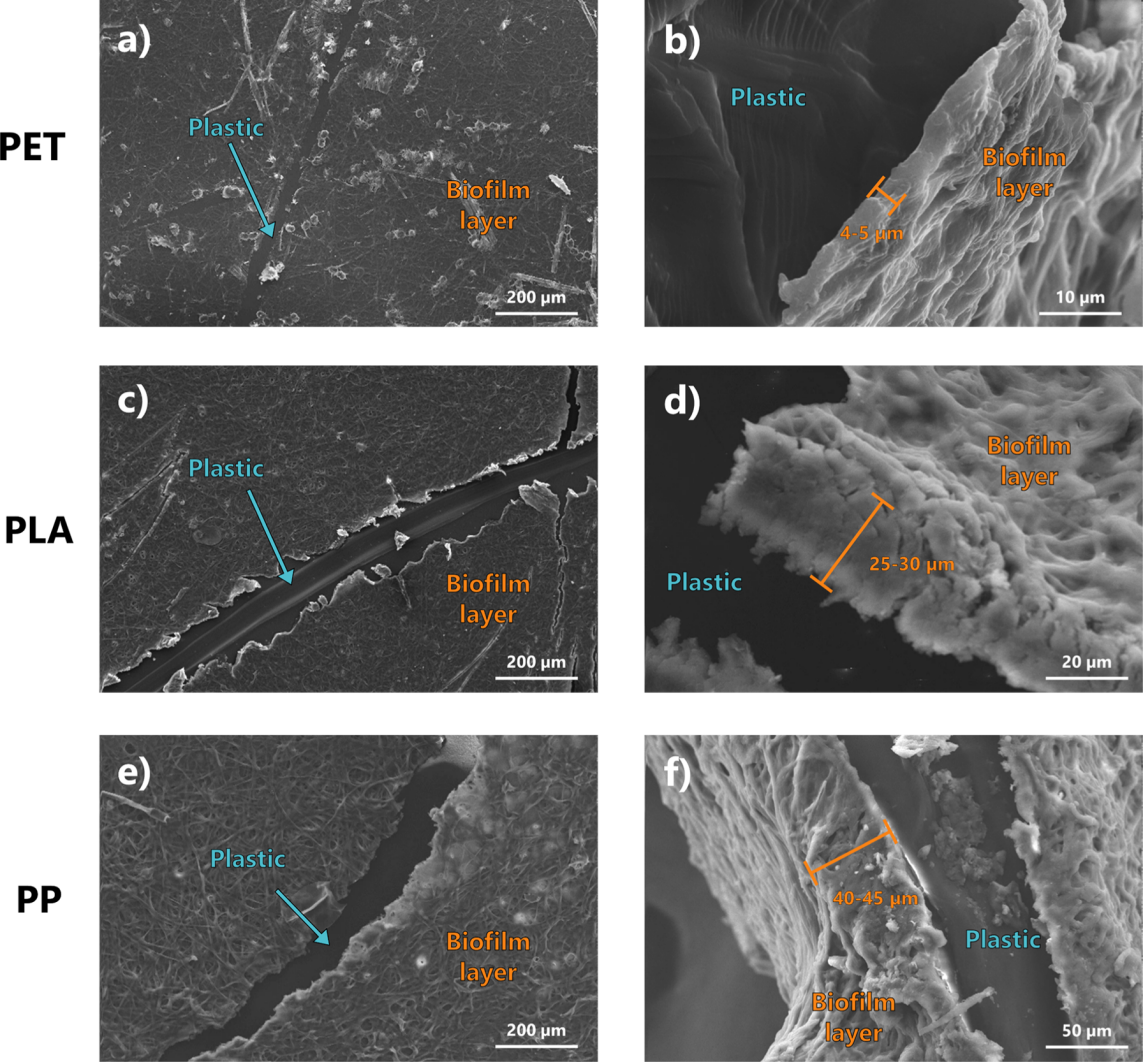
SEM micrograph of PET (panels a, b), PLA (panels c, d), and PP plastics (panels e, f) after the biotic aging process. Figures in the left column (panels a, c, and e) show the frontal view of the thick biofilm layer formed on the samples, while figures on the right column (panels b, d and f) show the lateral view, highlighting the different biofilm thickness.

Concerning the surface chemical properties, the IR spectra of all of the polymers showed the typical adsorption band of their composing polymers. For instance, the characteristic absorption bands located at 2960–2850 cm^–1^ and 1480–1300 cm^–1^ typical of C–H stretching of methyl and methylene groups for PP;^45^ the absorption peak of strong intensity at 1714 cm^–1^ (stretching of C=O of the carboxylic acid group) or the esters stretch at 1240 cm^–1^ for PET;^46,47^ and the band absorbing peaks at 1750 cm^–1^ and 1180 cm^–1^ related to stretching vibration of carbonyl (C=O) and ester (C–O) groups, as well as the peak at 1452 cm^–1^ assigned to bending of the –CH_3_ group for PLA^48,49^ (Figure 2). The formation of the biofilm altered the prevalence of surface functional groups regardless of the polymer type. For example, amide I and amide II bands are evident at 1650 cm^–1^ and 1550 cm^–1^, respectively, in almost all the polymers after aging (Figure 2). These peaks also altered the representative peak at 1714 cm^–1^ of PET, which showed a marked shoulder after aging (Figure 2a). Similarly, changes in intensity and shape of the OH band at 3500 cm^–1^ were observed in all the polymers: the broad peak in the 3000–3500 cm^–1^ band results in a sharper profile after aging, as an index of the presence of free and bonded hydroxyl groups and structural hydroxyl groups (–COOH and—COH).^50^ This is typical of the cell membranes and the extracellular polymeric substances associated with freshwater microorganisms, such as microalgae.^51^ These changes are clearly attributable to the biofilm formation, especially if we consider the depth probed by the analysis through ATR-IR measurements, which probe the first few micrometers below the sample surface. As the thickness of the biofilm layer observed from the SEM measurements is in the range of about 5 to 45 μm (as observable in Figure 1), the IR spectra are predominantly affected by the presence of the functional groups of the biofilm layer. The analysis of the EPS content also confirmed such changes induced by the presence of these compounds in the biofilm matrix (Figure S2).

**Figure 2:**
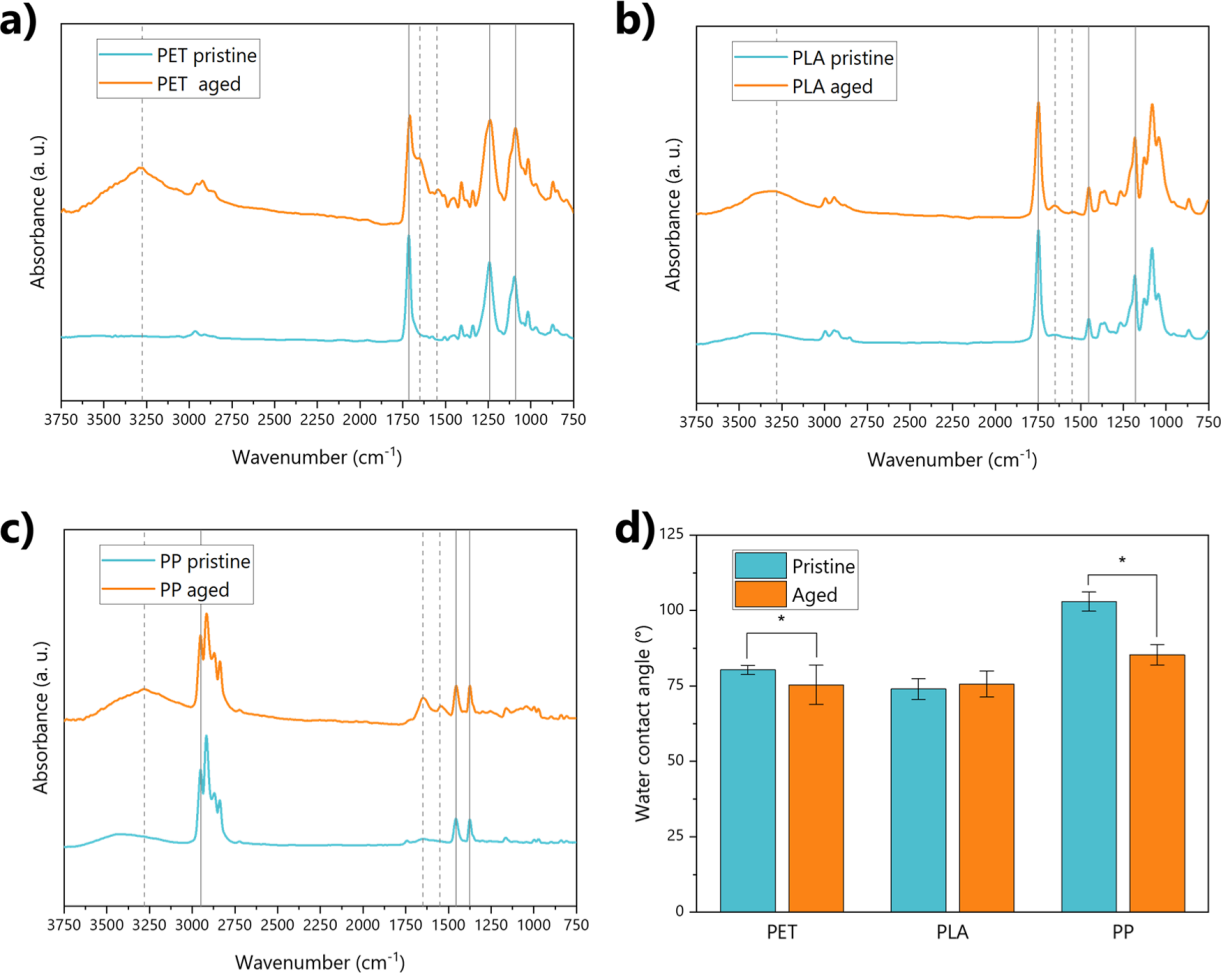
Physicochemical properties of pristine and aged plastics. Panels a, b, and c show the IR spectra of PET, PLA, and PP, respectively, before and after aging. Gray lines highlight the key functional groups of the polymers (if solid) and the functional groups affected by aging (if dashed). Panel d shows instead the values of water contact angles for all polymer samples. Significantly different values between pristine and aged plastics are highlighted by asterisks.

The changes observed in the functional groups were mirrored by changes in surface hydrophobicity through water contact angle measurements (Figure 2d). In general, biofilm formation induced a decline in hydrophobicity for PP and PET. In contrast, this effect was less pronounced for PLA, showing no significant change. It should be noted, however, that this polymer is inherently more hydrophilic than PP and PET in their pristine form. These changes in functional groups and the increased hydrophilicity are key parameters in enhancing the sorption capacity of metals for plastic fragments in water.^28–30^

### 3.2 Metal Content in Pristine and Aged Plastic

The total content of metals in the plastic samples (as analyzed through total sample digestion) is shown in Figure 3. Elements such as Sn and Sb present a clear trend along polymer composition, highlighting polymer-specific enrichment in PET and PLA. These metals instead showed values close to or below the LODs in samples composed of different polymers. Specifically, PET samples showed an Sb content of average 150 mg/kg, regardless of the samples being pristine or aged ones. This element was, instead, not detected in PLA and PP samples. Similarly, only PLA samples showed a Sn concentration of about 50 mg/kg, while Sn was not detected in PET and PP samples (Figure 3a and Table S3). These specific metals are then evidently concentrated in their respective polymers:^13,16^ their selective occurrence makes them ideal chemical markers as they are characteristic of a single polymer type and, thanks to their high concentrations in these polymer matrixes, they are easily detectable using conventional analytical techniques.^27^ This allows for straightforward identification and differentiation of polymer materials based on their metal signal in mixed or environmental samples and tracking of the plastic matrix–biofilm interface in cross-sectional analyses performed with LA–ICP–MS. Importantly, nonsignificant differences were detected in the amount of Sb and Sn in the pristine and aged samples, confirming that the biotic aging does not affect their levels and that these metals are native of the polymer matrix.

**Figure 3:**
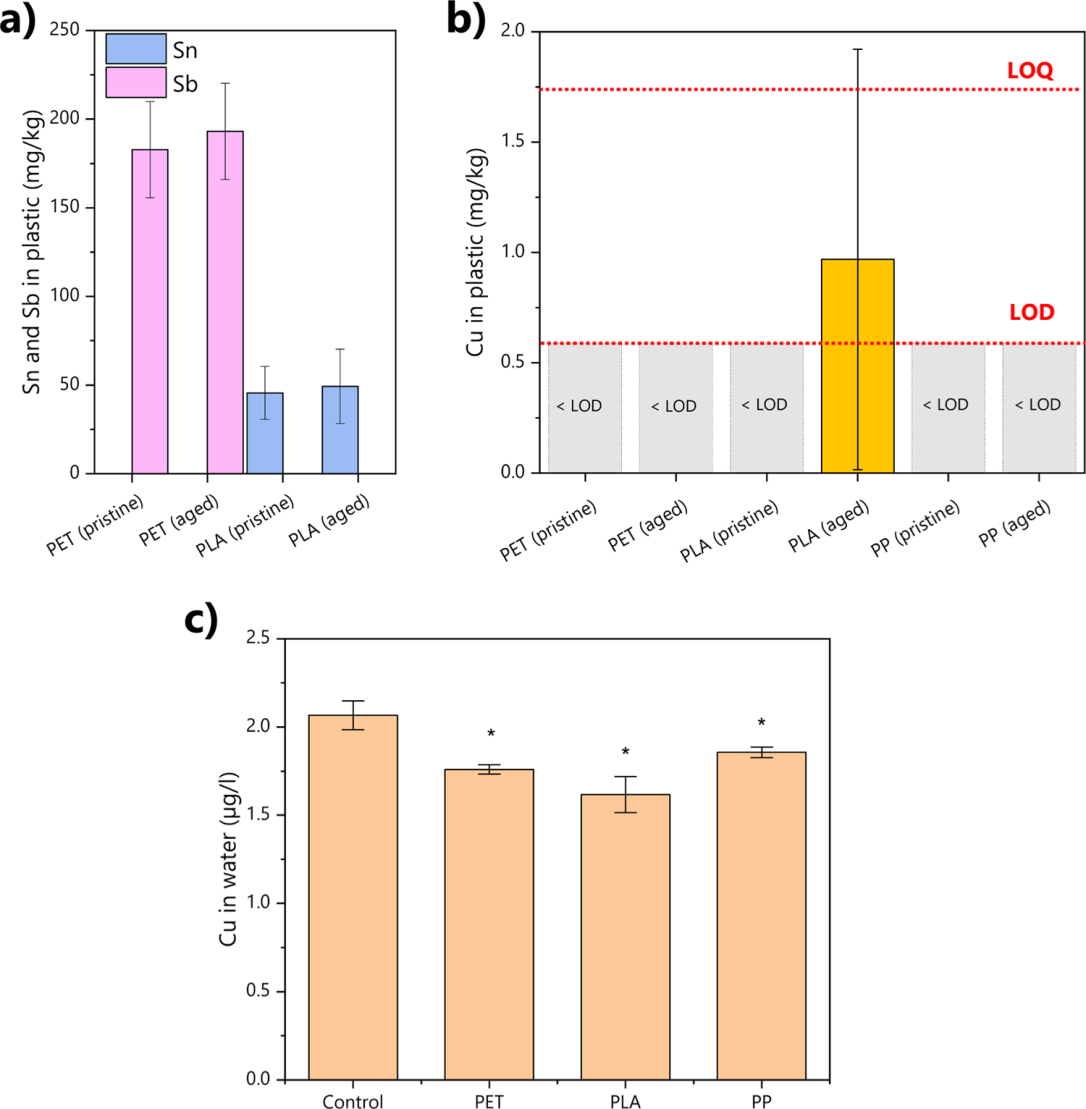
Concentration of metals in the different plastic samples and of Cu in water after 21 days of aging. Panel a shows the concentration of Sn and Sb in PET and PLA samples before and after aging, while PP data were below LODs and are not shown here (see Table S3 for details). Panel b shows the values of Cu concentrations after acid digestion in all samples, highlighting the LOQ and LOD for this element with red dashed lines. Panel c shows the concentration of dissolved Cu after 21 days, significantly different values from the control are highlighted with an asterisk, and time trends of Cu concentration in water are instead listed in Figure S4.

Copper results for total-digestion-based extraction showed a distinct and interesting behavior. This metal, in contrast to Sb and Sn, did not act as a specific marker of the polymer matrix. Instead, it accumulates from the water phase during aging, as shown by PLA results. However, Cu was below detection limits in pristine PLA and both pristine and aged PP and PET. This metal was detected only in PLA samples after aging, indicating a potential enrichment during the aging process. The analysis of Cu in the water solution during the aging process, analyzed weekly, showed in fact a significant decrease of Cu associated with the presence of plastic, regardless of the polymer type (Figure S4). While the growth of microorganisms caused a depletion of dissolved Cu concentration over time even without the presence of plastic in the control (i.e., water and algae without plastics), in all the plastic treatments depletion was significantly higher: after a notable depletion after 7 days of aging in all the treatments, likely induced by the initial growth phase of microorganisms in water and on the bottom of the Erlenmeyer glass flasks, the treatments containing plastic started to show a faster decrease in concentration, reaching averagely 15% less available Cu in solution at the end of the 21 day aging (Figure 3c). This evidence demonstrates that Cu is removed from the aquatic environment and is accumulated on the plastic samples.^28,29^ On the other hand, analysis through acid digestion followed by ICP–MS (the typical approach used to study trace elements in polymeric matrices) was unable to track this enrichment and close the Cu mass balance in the system. Assuming that the entire excess of Cu depleted from the water in the plastic-containing batches was all sorbed on the plastic fragments, its concentration in the bulk plastic samples presents values close to or below the LOD of the digestion method. In addition, some of the Cu may be also internalized in the organisms developed in the water phase as well as on the container walls during the aging, further complicating the quantification. The common approach of acid digestion seems, therefore, unsuited to detect relatively small variation in the accumulation of this metal on the surface of plastics. This points to a serious limitation of this commonly used technique to track metal accumulation on aged plastics from water environments.

### 3.3 Elemental Cross-Sectional Profiling in Plastic Samples

Having demonstrated the limitations of acid digestion coupled with ICP–MS for tracing biofilm effects on adsorption processes, we opted for a surface-sensitive technique, LA–ICP–MS, to selectively sample and analyze both the biofilm and the underlying polymer matrix.

To obtain a quantifiable depth profile with the LA–ICP–MS system, we first performed a preliminary optimization by varying fluence and spot size (Figure S5).^52^ SEM images revealed a limited partial fusion of the polymer matrix (Figure S6), which did not hinder the measurements; indeed, appreciable and reproducible signals were obtained for all of the materials investigated under the optimized conditions (Figure S7).

Next, we focused on the ablation depth. This parameter was found to be strongly polymer-dependent, with crater depths ranging from about 27 μm in PET to about 55 μm in PLA after five consecutive ablations (corresponding to ∼5.4 to 11 μm of material removed per laser shot, Figure S6). These measurements provided a solid basis to estimate the amount of the biofilm and plastic ablated at each step, allowing us to approximate the effective depth sampled in the polymer after every ablation event and, in turn, to construct a reliable elemental depth distribution (Figure 4).

**Figure 4:**
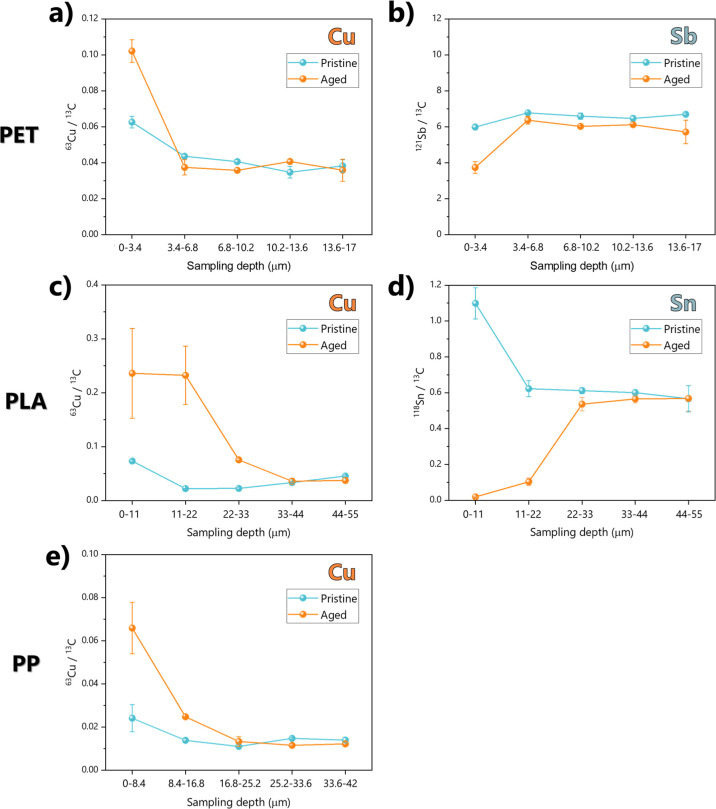
Metal concentration profiles on different plastic samples, at different depth intervals calculated for every ablation spot, depending on ablation efficiency of the polymer (see Figures S4 and S5). Panels a, c, and e show the trends for Cu concentration as a marker of biofilm-accumulated elements, while panel b shows the trend of Sb in PET, and panel d shows the trend of Sn in PLA (PET in panels a and b, PLA in panels c and d, and PP in panel e). In all panels, aged samples are depicted in orange and pristine samples are depicted in light blue.

The results with working parameters optimized for polymer-specific ablation are summarized in Figure 4 and detailed in Tables S4–S7. As a first clear observation, the depth-resolved profiles of Cu concentration in all aged samples revealed a markedly higher content in the outermost layers (within the first few tens of micrometers), which progressively decreased to values comparable to those found in pristine samples. This behavior highlights the extent of Cu enrichment at the surface compared to its distribution in the polymer bulk, in agreement with previous evidence analyzing environmentally collected plastic samples.^21,22,25^ Such behavior was observed irrespective of the polymer type, further supporting the hypothesis that Cu adsorption is primarily a biofilm-mediated process.^12,28^ The thickness of the Cu-enriched layer observed by LA–ICP–MS is consistent with the thickness of the biofilms observed by SEM pictures (Figure 1). For instance, surface cross-sections of the PET profile show a sharp decrease of the Cu signal, reaching a concentration comparable with the pristine signal (i.e., the bulk polymer) after one ablation (sampling the depth between 0 and 3.4 μm), while for the other two polymers, the signals converge to a value comparable to the pristine polymer one after 2 ablations, at about 20 μm ablation depth. The Cu signal in the biofilm layer of PLA (Figure 4c) was about 10-fold higher than in the other plastic types, reflecting results from acid digestions (Figure 3b). In addition, the depletion of dissolved Cu in the water phase of the PLA treatments was higher than for other polymers, further confirming enhanced biofilm growth and Cu sequestration on PLA (Figures 3c and S2). These results collectively confirm that Cu can serve as a reliable chemical marker of the biofilm layer.

On the other hand, the elements that were systematically abundant in specific polymer types (i.e., Sn in PLA and Sb in PET) exhibited negligible signals in the outermost layers of the surficial cross-section of aged samples, with sharply increasing values approaching those measured in the bulk plastic concentrations of pristine materials as the ablation depth reached the surface of plastics.^24,25^ The cross-sectional profiles of Sb and Sn display the opposite behavior of that observed for Cu, providing an additional indication of the presence and thickness of the biofilm layer, selectively accumulating Cu from the water solution. Therefore, while Cu can serve as a chemical marker for biofilm and its thickness, Sn and Sb act as markers of the bulk polymer for PLA and PET, respectively. These complementary signals can therefore be used for an accurate assessment of the biological aging of particles. Such conclusions cannot instead be clearly drawn for our PP samples, as in the PP materials used here we could not find any specific metal markers. In this case, only the biofilm marker of Cu can be used to trace the presence of the biofilm (Figure 4e). This evidence further supports the unique value of polymer-specific chemical markers in this type of investigation.

As shown in Figure 4, LA–ICP–MS analysis shows that pristine plastics may also exhibit uneven metal distribution in surface cross-sections: significantly higher concentrations of Cu and Sn are observed in the very outermost layer (i.e., during the first ablation step) in comparison to the bulk polymer material, most likely resulting from surface diffusion of these species within the polymer matrix during the manufacturing of the plastic objects.^4,25,53^ However, the combined use of the biofilm and bulk polymer markers allows us to disambiguate between polymer diffusion and biofilm adsorption processes as they should show an opposite depth-resolved trend.

### 3.4 Advantages, Limits, and Future Developments for the Application of LA–ICP–MS

This study showcases the potential of LA–ICP–MS to complement common techniques to investigate the interactions of plastics and metals in aquatic ecosystems. This technique enabled us to highlight the marked enrichment in Cu on the surface of aged plastic, in comparison to pristine one, associated with the biofilm. A key advantage of LA–ICP–MS is its surface sensitivity combined with high analytical sensitivity.^21,23,41^ In contrast, the traditional acid digestion protocol failed to quantify adsorbed species, as evidenced by Cu values consistently below the limit of quantification (Figure 3).

Depth-resolved capability of LA–ICP–MS enabled the distinction between biofilm-associated metal signals and bulk polymer contributions, providing also a quantitative estimation of biofilm thickness. By simultaneously monitoring biofilm markers (e.g., Cu) and polymer-specific additives (e.g., Sn in PLA, Sb in PET), the technique offers an array of complementary information to unambiguously discriminate between metal adsorption from the environment and additive diffusion within the polymer matrix: such a result is not achievable using conventional techniques. In other words, our results demonstrate that LA–ICP–MS can serve as a chemical marker-based approach particularly valuable for assessing surface versus bulk metal distributions, which is critical for understanding the environmental fate of specific elements in the context of widespread plastic contamination. This study represents a first step in this type of investigation, highlighting an approach with considerable potential that has so far been largely overlooked in the current literature.^24^

Nonetheless, there are some limitations to consider and future refinements to improve the applicability of this technique. The technique requires careful optimization of instrumental parameters (e.g., fluence, spot size, number of ablations) to avoid polymer melting or irreproducible ablation, and quantitative interpretation can be influenced by polymer-specific behavior.^54,55^ Moreover, for polymers lacking systematically present metal additives (such as PP in this study), only biofilm markers (e.g., Cu) can be used to trace biofilm presence, limiting the ability to simultaneously monitor bulk polymer contributions. Additionally, for a more precise quantification of metals in the polymer matrix, the use of standard reference materials would be highly beneficial to calibrate ablation efficiency and account for matrix effects.^52,56^ However, certified plastic materials with known trace element content are not available for many polymer types, and importantly, only their bulk content is known, with no information on the distribution of elements within the polymer matrix at the microscopic scale.^32^ This information is crucial for LA–ICP–MS analysis.^54^

A further step to assess the reliability of this approach for investigating plastic–metal interactions and the key role of biofilms in mediating these processes would be to compare LA–ICP–MS with other high-resolution surface techniques capable of chemical mapping and depth profiling in these matrices, such as secondary ion mass spectrometry (SIMS) or synchrotron-based X-ray techniques.^57,58^ Although these methods are substantially more costly and require more demanding sample handling and preparation, they offer higher spatial resolution and analytical sensitivity, complementing the insights obtained by LA–ICP–MS. In addition, while being less sensitive for specific metal markers, Raman microscopy may also be applied as a comparative approach to investigate biofilm-mediated environmental processes at plastic surfaces.^59^

## 4 Conclusions

Understanding the mechanisms by which plastics interact with metals is essential for properly evaluating their chemical risks, and LA–ICP–MS can offer a valuable tool to complement conventional characterization methods. In this study, we tested the feasibility of this approach under controlled laboratory conditions, using three different polymer types subjected to biotic aging and focusing on a set of metals to be used as markers of the biofilm (i.e., Cu) and of the polymer matrix (i.e., Sb and Sn). Our LA–ICP–MS results showed the marked accumulation of Cu from water on the biofilm layer covering aged plastics on all polymer types. This was confirmed by the marked decrease in Cu in the water phase, indicating its potential sorption. By contrast, conventional acid digestion revealed only limited amounts of Cu in the plastic samples, indicating the superior and unique performances of LA–ICP–MS in understanding plastic and metal interactions. The use of metallic chemical markers for plastic also proved useful in interpreting the patterns of metal enrichment associated with biofilm development, presenting a high potential for environmental applications. In summary, this study demonstrates the potential of LA–ICP–MS as a powerful technique for investigating plastic–metal interactions in controlled settings and highlights, for the first time, the combined use of polymer-specific markers and depth-resolved analyses to better understand metal enrichment processes driven by biofilms. This technique represents a promising surface-sensitive approach that can fill gaps left by conventional techniques. It provides unique insights into the spatial distribution of metals on and within plastic materials, enabling the study of biofilm-mediated sorption processes under environmentally relevant conditions.

## Supplementary Material



## Notes

Additional figures and tables on the formation and physicochemical properties of the aged plastics, metal content in water and plastic in the aging batches, and LA–ICP–MS performances and setup (PDF)
