# Supplementary material for: Investigating Plastic–Metal Interactions in Aquatic Environments Using Laser Ablation ICP–MS and Chemical Markers
Source: ACS ES T Water. 2026 Feb 18;6(3):1925–35. doi: 10.1021/acsestwater.5c01387 (PMC12993839; doi:10.1021/acsestwater.5c01387)
Supplement: Supplementary file 1 [file ew5c01387_si_001.pdf]

Investigating Plastic–Metal Interactions in Aquatic Environments Using Laser Ablation ICP-MS and chemical markers

Davide Spanu[a], Ludovica Botta[a], Stefano Carnati[a], Tommaso Grande[b], Gabriela Kalčíková[c,d], Luca Nizzetto[e,f], Andrea Pozzi[a], Luka Šupraha[e], Gilberto Binda[b, e] *

[a] Department of Science and High Technology, University of Insubria, Via Valleggio 11, 22100 Como, Italy

[b] Department of Theoretical and Applied Science, University of Insubria, via J.H. Dunant 3, 21100 Varese, Italy

[c] Faculty of Chemistry and Chemical Technology, University of Ljubljana, Večna pot 113, Ljubljana 1000, Slovenia

[d] Faculty of Mechanical Engineering, Brno University of Technology, Technická 2896/2, Brno 61669, Czech Republic

[e] Norwegian Institute for Water Research (NIVA), Økernveien 94, 0579 Oslo, Norway

[f] RECETOX, Masaryk University, Kamenice 753/5, 625 00 Brno, Czech Republic

*Corresponding author, email address: gilberto.binda@uninsubria.it

Supporting Information

Figures S1-S7

Tables S1-S7

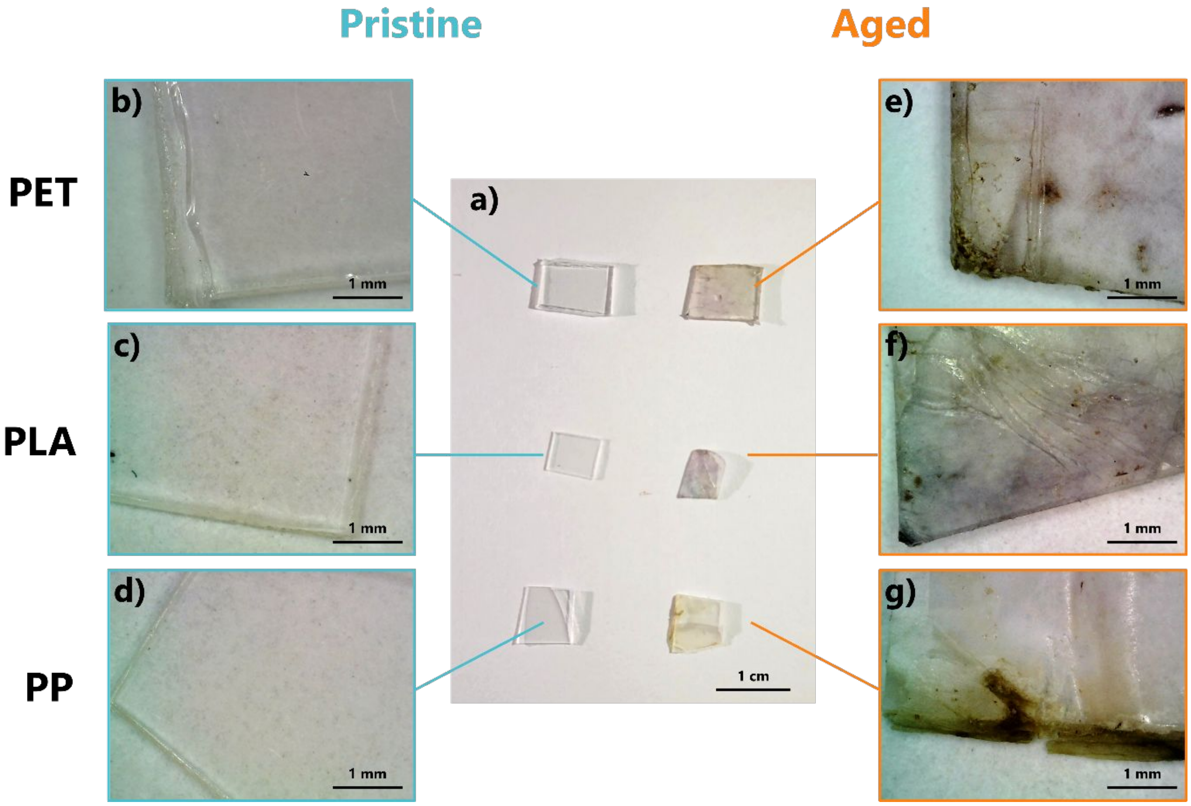

Figure S1. Panel a: photographs of all polymers in pristine (on the left) and after aged form on the right. All the aged samples show an evident and homogenous biofilm covering the plastic polymer. Panels b, c and d show details of pristine PET, PLA and PP, while panels e, f and g show same magnifications for their aged form. This further confirms an evident change in sample morphology.

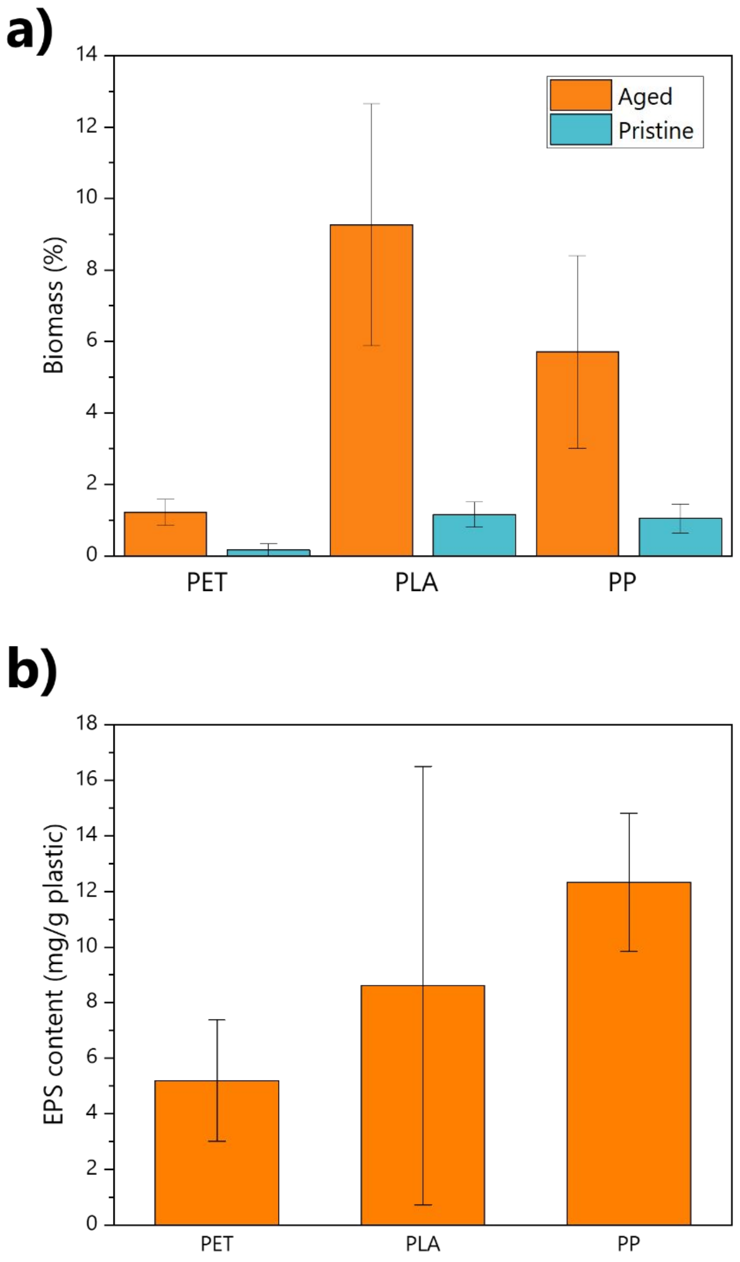

Figure S2. a) Biofilm biomass expressed in percentage of the total mass of the plastic fragments after Fenton oxidation of aged (in orange) and pristine (in light blue) plastic. The mass loss of pristine plastic was computed to assess the potential polymer degradation by Fenton digestion. b) Extracellular polymeric substances (EPS) concentrations in the different aged polymers. Results are shown as average ± standard deviation after three replicates.

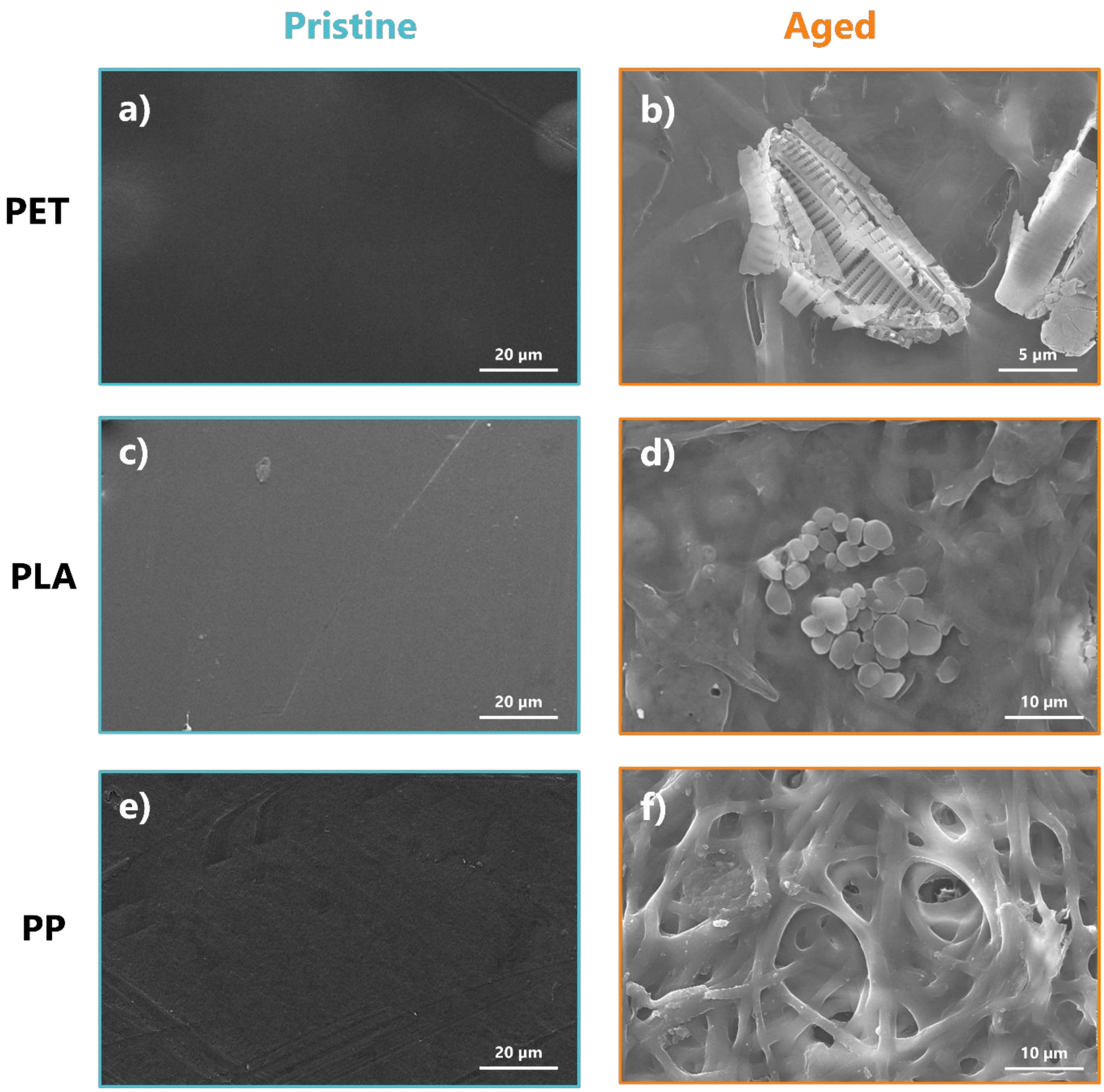

Figure S3. SEM micrographs of all polymers before (panels a, c and e) and after biotic aging (panels b, d and f). Several microorganisms are visible after aging, such as diatom frustules (panel b), coccoids (panel e) and a thick cover o filamentous algae (panel f).

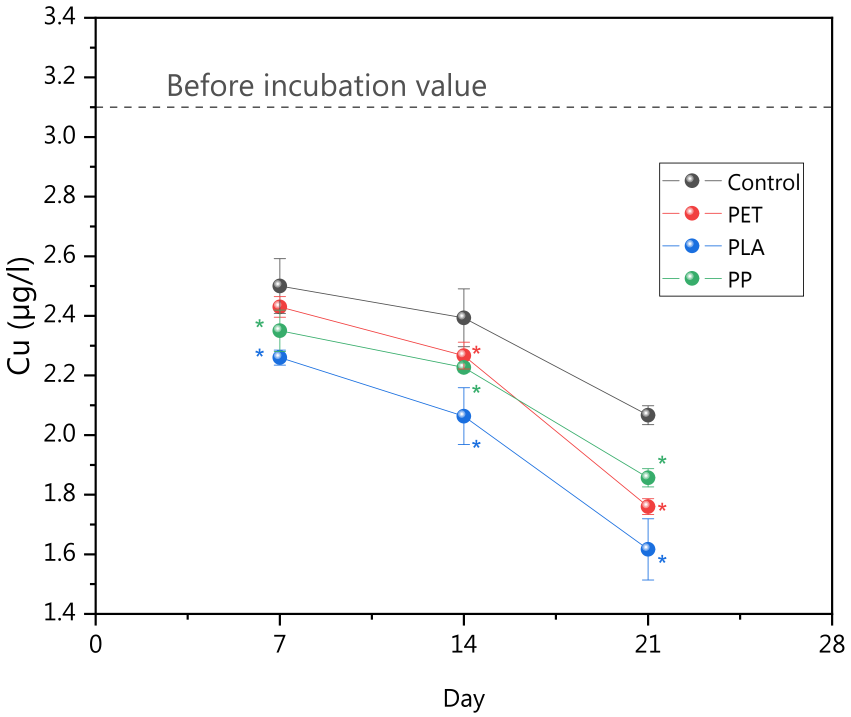

Figure S4. Time trend of dissolved Cu concentrations in water (in µg/l) over the 21 days of incubation experiment in absence of plastic (control) and with the 3 different polymer types. Significant differences from the control after t-test are indicated with an asterisk, with different color indicating the polymer type. Dashed grey line indicates the concentration of Cu before the incubation experiment.

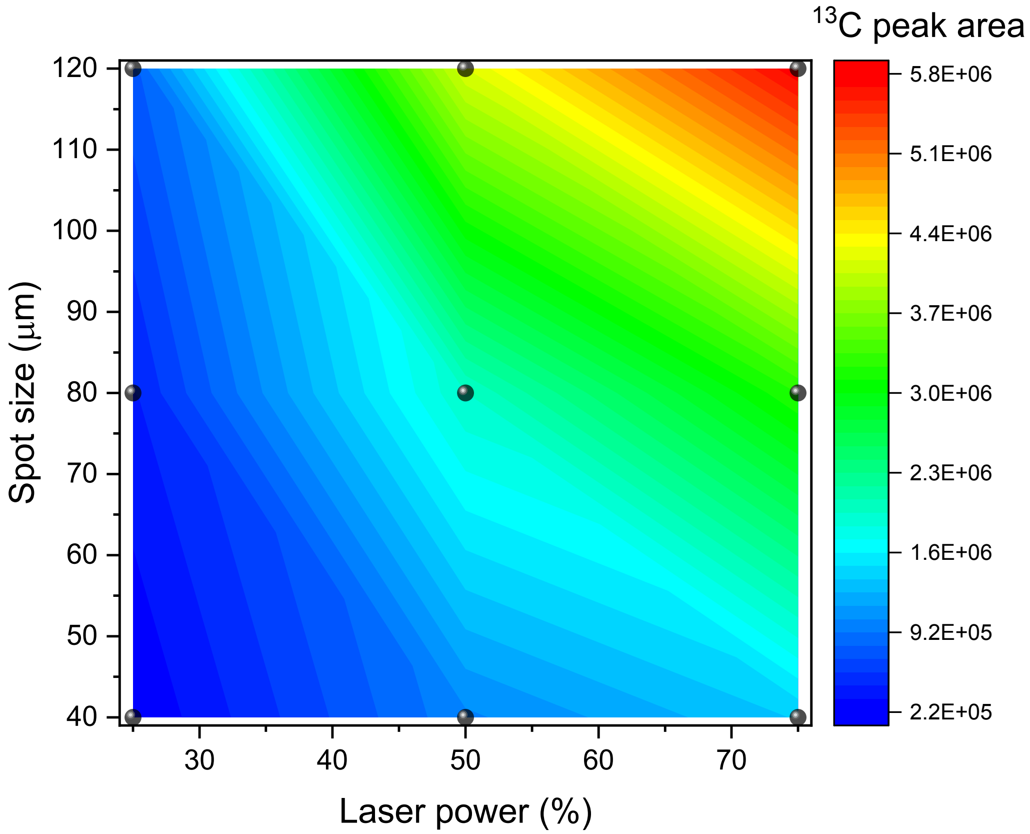

Figure S5. Contouring map reporting the intensity of the signal on channel ^{13}C expressed as the peak area by varying the laser spot size and power. These signals refer to the analysis of a PET pristine sample. Black circles represent the tested conditions. The following conditions were chosen as the optimum in terms of sensitivity: laser spot size = 120 µm, laser power = 75% (equal to a fluence of 6.7 J/cm^2).

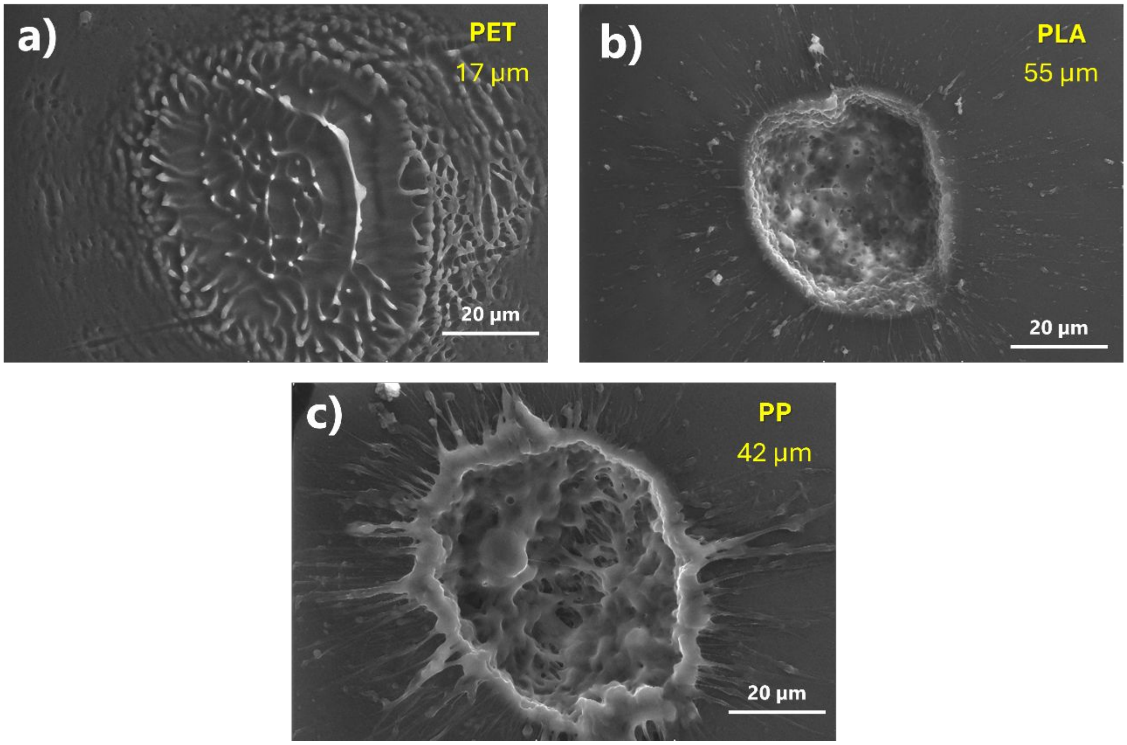

Figure S6. SEM micrographs of the craters formed after 5 consecutive ablations on the different polymer samples (PET in panel a, PLA in panel b and PP in panel c). Estimated depth of the craters is also shown in the upper-right corner of every panel.

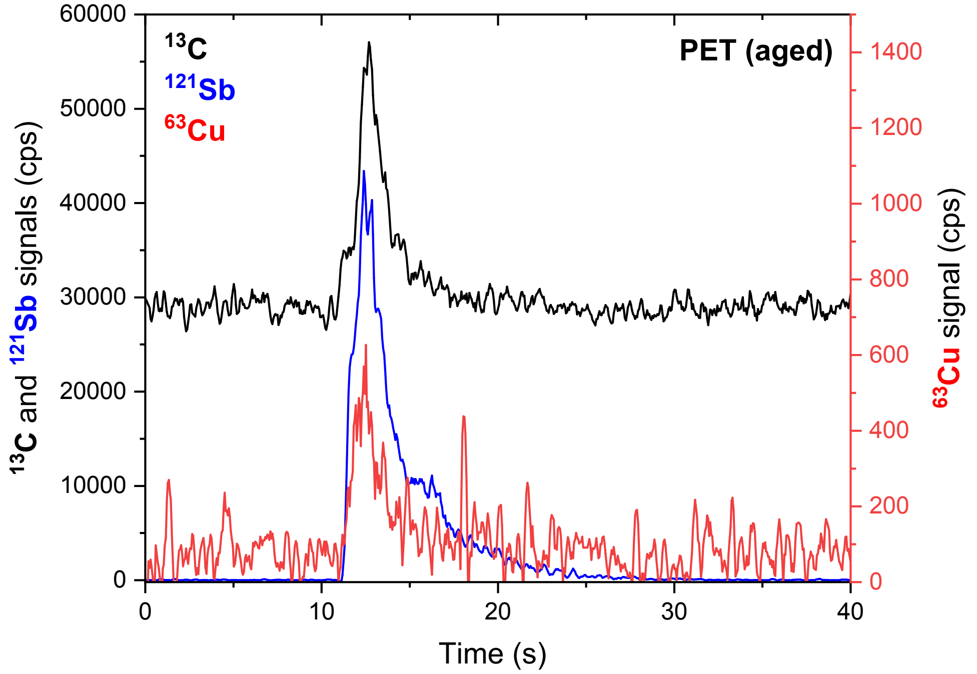

Figure S7. LA-ICP-MS signals obtained after the first ablation on a PET (aged) sample under optimized laser conditions.

Table S1. Physicochemical parameters of the pond water selected to sample the microbial community, measured after sampling in March 2025.

Feature	Measure unit	Value
Temperature	˚ C	16±0.2
pH	-	9.7±0.05
Conductivity	µS/cm	187±2
Alkalinity	mmol/l	1.25±0
Ca	mg/l	24.04±0.48
K	mg/l	2.3±0.05
Mg	mg/l	3.21±0.06
Na	mg/l	21.74±0.43
F	mg/l	0.39±0.02
Cl	mg/l	30.6±1.53
NO_2	mg/l	<0.05
Br	mg/l	0.19±0
NO_3	mg/l	<0.05
PO_4	mg/l	<0.05
SO_4	mg/l	16.45±0.33
Cu	µg/l	5.71±0.23
Sn	µg/l	0.061±0
Sb	µg/l	0.579±0.02

Table S2. Working parameters of the ICP-MS instrument for the measurements of both laser ablation, waters samples and digested plastic samples.

Parameters	Value
RF power (kW)	1.55
Auxiliary gas flux (L/min)	0.8
Cooling gas flux (L/min)	14
Nebulizer gas flux (L/min)	1.2
Dwell time (ms)	10

Table S3. Acid digestion data (indicated as average ± standard deviation after 3 measure replicates). Limit of detections (LODs) and of quantifications (LOQs) are also shown, estimating a sample mass of 100 mg.

Sample	Cu mg/kg	Sn mg/kg	Sb mg/kg
PET (pristine)	<LOD	0.038±0.017	183±27.15
PET (aged)	<LOD	0.232±0.291	193±35.5
PLA (pristine)	<LOD	45.6±14.861	2.08±2.76
PLA (aged)	0.968±0.953	49.3±20.992	0.128±0
PP (pristine)	<LOD	0.061±0.05	0.128±0
PP (aged)	<LOD	0.06±0.049	1.252±1.301
LOD	0.587	0.051	0.256
LOQ	1.761	0.153	0.768

Table S4. Peak areas obtained for investigated elements in pristine and aged PET by LA-ICP-MS.

PET (pristine) – Replicate #1					
**Isotope**	**1st ablation**	**2nd ablation**	**3rd ablation**	**4th ablation**	**5th ablation**
^{13}C	36169.4	33785.6	34033.2	35597.7	33001.5
^{63}Cu	2102.3	1529.4	1434.2	1399.6	1422.8
^{118}Sn	n.d.	n.d.	n.d.	n.d.	n.d.
^{121}Sb	209245.4	228579.7	233015.6	238106.7	217628.3
**PET (pristine) – Replicate #2**					
**Isotope**	**1st ablation**	**2nd ablation**	**3rd ablation**	**4th ablation**	**5th ablation**
^{13}C	40211.9	33731.9	38208.2	39500.7	36489.3
^{63}Cu	2701.7	1419.9	1497.6	1195.2	1221.3
^{118}Sn	n.d.	n.d.	n.d.	n.d.	n.d.
^{121}Sb	248579.6	229293.2	242612.1	247077.7	248283.1
**PET (aged) – Replicate #1**					
**Isotope**	**1st ablation**	**2nd ablation**	**3rd ablation**	**4th ablation**	**5th ablation**
^{13}C	38880.7	38450.7	41294.9	41040.8	33906.6
^{63}Cu	4317.3	1673.2	1480.3	1595.5	1515.0
^{118}Sn	n.d.	n.d.	n.d.	n.d.	n.d.
^{121}Sb	128035.6	233594.3	251139.8	252528.2	225150.8
**PET (aged) – Replicate #2**					
**Isotope**	**1st ablation**	**2nd ablation**	**3rd ablation**	**4th ablation**	**5th ablation**
^{13}C	53667.8	35811.1	41932.1	41222.1	54316.4
^{63}Cu	5008.4	1126.2	1503.2	1756.8	1477.4
^{118}Sn	n.d.	n.d.	n.d.	n.d.	n.d.
^{121}Sb	225009.0	238946.3	250557.9	251232.0	260308.0

Table S5. Peak areas obtained for investigated elements in pristine and aged PLA by LA-ICP-MS.

PLA (pristine) – Replicate #1					
**Isotope**	**1st ablation**	**2nd ablation**	**3rd ablation**	**4th ablation**	**5th ablation**
^{13}C	27461.4	59269.3	63869.4	37719.8	25328.6
^{63}Cu	1929.6	1322.0	1249.9	1324.9	1221.2
^{118}Sn	33386.6	33209.3	37555.7	21750.5	16982.1
^{121}Sb	n.d.	n.d.	n.d.	n.d.	n.d.
**PLA (pristine) – Replicate #2**					
**Isotope**	**1st ablation**	**2nd ablation**	**3rd ablation**	**4th ablation**	**5th ablation**
^{13}C	24514.6	52971.7	48713.6	38281.1	29990.7
^{63}Cu	1872.2	1154.9	1244.2	1209.7	1284.6
^{118}Sn	23937.4	36296.6	30984.2	23903.3	13884.7
^{121}Sb	n.d.	n.d.	n.d.	n.d.	n.d.
**PLA (aged) – Replicate #1**					
**Isotope**	**1st ablation**	**2nd ablation**	**3rd ablation**	**4th ablation**	**5th ablation**
^{13}C	59727.6	33590.4	41541.4	50182.1	45179.6
^{63}Cu	21142.0	10376.4	2943.5	2165.7	2004.5
^{118}Sn	1215.3	4479.0	20142.2	26902.4	30209.1
^{121}Sb	n.d.	n.d.	n.d.	n.d.	n.d.
**PLA (aged) – Replicate #2**					
**Isotope**	**1st ablation**	**2nd ablation**	**3rd ablation**	**4th ablation**	**5th ablation**
^{13}C	78662.0	44169.3	36591.5	69722.8	61132.6
^{63}Cu	9296.9	6887.2	2934.8	1993.1	1851.9
^{118}Sn	1419.7	3257.5	21514.8	41504.8	28611.2
^{121}Sb	n.d.	n.d.	n.d.	n.d.	n.d.

Table S6. Peak areas obtained for investigated elements in pristine and aged PP by LA-ICP-MS.

PP (pristine) – Replicate #1					
Isotope	1st ablation	2nd ablation	3rd ablation	4th ablation	5th ablation
^{13}C	39167.1	76495.8	100692.2	77924.8	67764.1
^{63}Cu	716.2	1149.1	1008.0	1091.6	1065.7
^{118}Sn	n.d.	n.d.	n.d.	n.d.	n.d.
^{121}Sb	n.d.	n.d.	n.d.	n.d.	n.d.
PP (pristine) – Replicate #2					
Isotope	1st ablation	2nd ablation	3rd ablation	4th ablation	5th ablation
^{13}C	42709.2	83960.1	104514.2	73384.1	84682.3
^{63}Cu	1276.1	1065.7	1261.5	1143.4	1036.9
^{118}Sn	n.d.	n.d.	n.d.	n.d.	n.d.
^{121}Sb	n.d.	n.d.	n.d.	n.d.	n.d.
PP (aged) – Replicate #1					
Isotope	1st ablation	2nd ablation	3rd ablation	4th ablation	5th ablation
^{13}C	43710.3	54099.4	70920.1	89946.3	102901.4
^{63}Cu	2142.7	1336.3	1172.3	1128.9	1126.2
^{118}Sn	n.d.	n.d.	n.d.	n.d.	n.d.
^{121}Sb	n.d.	n.d.	n.d.	n.d.	n.d.
PP (aged) – Replicate #2					
Isotope	1st ablation	2nd ablation	3rd ablation	4th ablation	5th ablation
^{13}C	46464.3	48346.1	92116.3	115865.0	87327.6
^{63}Cu	3845.3	1203.8	936.1	1229.8	1178.1
^{118}Sn	n.d.	n.d.	n.d.	n.d.	n.d.
^{121}Sb	n.d.	n.d.	n.d.	n.d.	n.d.

Table S7. Average peak areas normalized to the ^{13}C internal standard signal for detectable elements in Tables S4-S6. Uncertainties are reported as one standard deviation (n = 2). Sampling depth was estimated by SEM (see Section 2.6 in the main text).

PET (pristine)		
**Sampling depth (μm)**	**$^{63}Cu/^{13}C$**	**$^{121}Sb/^{13}C$**
0 - 3.4	0.0627 ± 0.0032	5.98 ± 0.14
3.4 - 6.8	0.0437 ± 0.0011	6.78 ± 0.01
6.8 – 10.2	0.0407 ± 0.001	6.6 ± 0.18
10.2 – 13.6	0.0348 ± 0.0032	6.47 ± 0.15
13.6 - 17	0.0383 ± 0.0034	6.7 ± 0.07
**PET (aged)**		
**Sampling depth (μm)**	**$^{63}Cu/^{13}C$**	**$^{121}Sb/^{13}C$**
0 - 3.4	0.1022 ± 0.0063	3.74 ± 0.32
3.4 - 6.8	0.0375 ± 0.0043	6.37 ± 0.21
6.8 – 10.2	0.0358 ± 0.0001	6.03 ± 0.04
10.2 – 13.6	0.0407 ± 0.0013	6.12 ± 0.02
13.6 - 17	0.0359 ± 0.0062	5.72 ± 0.65
**PLA (pristine)**		
**Sampling depth (μm)**	**$^{63}Cu/^{13}C$**	**Sn/C**
0 - 11	0.0733 ± 0.0022	1.096 ± 0.085
11 - 22	0.0221 ± 0.0002	0.623 ± 0.044
22 - 33	0.0226 ± 0.0021	0.612 ± 0.017
33 - 44	0.0334 ± 0.0012	0.601 ± 0.017
44 - 55	0.0455 ± 0.0019	0.567 ± 0.073
**PLA (aged)**		
**Sampling depth (μm)**	**$^{63}Cu/^{13}C$**	**Sn/C**
0 - 11	0.2361 ± 0.0834	0.019 ± 0.001
11 - 22	0.2324 ± 0.0541	0.104 ± 0.021
22 - 33	0.0755 ± 0.0033	0.536 ± 0.036
33 - 44	0.0359 ± 0.0052	0.566 ± 0.021
44 - 55	0.0373 ± 0.005	0.568 ± 0.071
	**PP (pristine)**	**PP (aged)**
**Sampling depth (μm)**	**$^{63}Cu/^{13}C$**	**$^{63}Cu/^{13}C$**
0 – 8.4	0.0241 ± 0.0041	0.0659 ± 0.0119
8.4 – 16.8	0.0139 ± 0.0008	0.0248 ± 0.0001
16.8 - 25.2	0.011 ± 0.0007	0.0133 ± 0.0023
25.2 – 33.6	0.0148 ± 0.0006	0.0116 ± 0.0007
33.6 - 42	0.014 ± 0.0012	0.0122 ± 0.0009
